# The aging ovary stands on the shoulders of giant multinucleated cells

**DOI:** 10.1371/journal.pbio.3003216

**Published:** 2025-06-24

**Authors:** Avery A. Ahmed, Stephanie A. Pangas

**Affiliations:** 1 Department of Pathology & Immunology, Baylor College of Medicine, Houston, Texas, United States of America; 2 Graduate Program in Development, Disease Models & Therapeutics, Baylor College of Medicine, Houston, Texas, United States of America; 3 Department of Molecular & Cellular Biology, Baylor College of Medicine, Houston, Texas, United States of America

## Abstract

Reproductive aging is associated with declining fertility and increasing inflammation, though these events are not well understood. This Primer discusses a new study that uses cutting-edge technologies to characterize the role of multinucleated giant cells in ovarian aging.

## Teaser

Reproductive aging is associated with declining fertility and increasing inflammation, though these events are not well understood. An exciting new study in *PLOS Biology* utilizes cutting-edge technologies to characterize the role of multinucleated giant cells in ovarian aging.

Ovarian aging is associated with a reduction in both the quantity and quality of oocytes, leading to a general decline in female fertility with age [1]. Reproduction ends at the menopause in humans, but other mammals also show reproductive decline with age [2]. The oocyte develops in tandem with somatic cells of the ovarian follicle, which are surrounded by the ovarian stroma. How changes to the ovarian stroma that occur with disease and aging impact follicle development and fecundity is an increasing area of research interest. One well-documented phenomenon in aging tissues, including the ovary, is increasing inflammation over time, a concept referred to as “inflammaging” [3]. Though recent studies indicate ovarian fibrosis, a marker of inflammaging, can be therapeutically targeted in aging mice to improve fertility [4], specific events driving inflammaging have not been well characterized.

A unique type of immune cell, multinucleated giant cells (MNGCs), are associated with a range of inflammatory responses [5]. MNGCs are absent in young mouse ovaries but present in aged mouse ovaries [6]. However, their characterization in the ovary and other somatic tissues has been limited by technical challenges. First, MNGCs are too large for size exclusion for current single-cell RNA sequencing protocols, and performing bulk tissue RNA sequencing complicates the determination of an MNGC-specific molecular signature. Further, MNGCs have been difficult to isolate from tissues. In vitro methods of induced macrophage fusion models are frequently used as substitutes for isolated MNGCs [7], but it is unclear how closely this recapitulates the in vivo environment of the tissue. These limitations have resulted in the lack of a comprehensive investigation into MNGC dynamics during aging.

**Fig 1:**
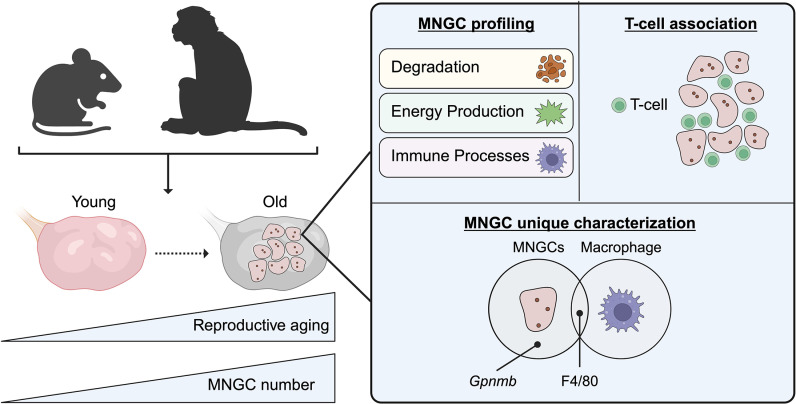
Multinucleated giant cells (MNGCs) proliferate with age and show unique transcriptomic profiles. In ovaries collected from mice and nonhuman primates (NHPs), MNGC penetrance increases with reproductive age (*left panel*). Transcriptomic profiling of MNGCs reveals the top gene categories as degradation, energy production, and immune processes (*right panel top left section*). Further, MNGCs are closely associated with T-cells (*right panel top right section*). Additional transcriptomic analysis segregates MNGCs from macrophages and stromal cells. While stromal cells serve as controls and do not overlap with macrophages or MNGCs, macrophages and MNGCs share expression of macrophage marker F4/80. However, the authors identify *Gpnmb* as a unique, putative marker of ovarian MNGCs (*right panel bottom section*). Created in BioRender: Pangas, S. (2025) https://BioRender.com/y3bfssy.

To address these issues, in a new study in PLOS Biology, Converse and colleagues employ cutting-edge technologies to characterize, isolate, and analyze the transcriptomic signature of MNGCs in the ovary [8]. The authors first investigated the penetrance of MNGCs in ovaries with age using young and aged mouse tissue as well as aged nonhuman primate (NHP) tissue (Fig 1). In both the aged mouse and NHPs, MNGCs are present, and their percentage of the total ovarian volume increases with age. Further, the authors mapped in 3D, MNGCs within their resident tissue taking advantage of their autofluorescent properties and using multiphoton microscopy. The 3D fluorescent images capture the large and complex networks of MNGCs within the aging ovary for the first time.

Once the overarching structure of the MNGC network was characterized, the question remained of how to isolate these large networks to perform molecular analyses. This imposed both technical and informatic challenges. Converse and colleagues overcame the size exclusionary difficulties of sequencing MNGCs by isolating enriched populations using laser capture microdissection (LCM) and using these samples for bulk RNA sequencing [8]. Transcriptomic profiling of LCM-isolated aged mouse ovary MNGCs reveals that the top 50 highly expressed genes indicate roles in “degradation”, immune system, and oxidative phosphorylation, confirming that MNGCs have an immune function and further suggests that MNGCs have upregulated energy production. The authors also identified *Gpnmb*, a transmembrane glycoprotein and putative marker of non-ovarian MNGCs, as specific to MNGCs in the mouse ovary, and its increased protein expression with age correlates with increased MNGCs. Thus, Converse and colleagues provide the first molecular profile of ovarian MNGCs and suggest *Gpnmb* as a putative marker of MNGCs in the ovary.

The transcriptomic data Converse and colleagues collected from mouse ovary MNGCs also allowed the authors to interrogate the origin of MNGCs in the ovary [8]. The authors speculated that MNGCs are at least partially derived from macrophages due to the expression of macrophage markers like F4/80; however, it remained unclear how MNGCs differ from macrophages. To answer this question, the authors collected young mouse macrophages by immunomagnetic pulldown of F4/80+ cells and performed RNA sequencing. Principal component analysis shows that macrophages and MNGCs do not cluster, suggesting that MNGCs are distinct from macrophages. Comparison of differentially expressed genes between MNGCs and macrophages shows that processes related to morphogenesis and differentiation are downregulated in MNGCs. The authors hypothesize from these data that MNGCs may be a ‘less plastic’ or a more differentiated version of macrophages. This presents a novel idea of how MNGCs may arise, which warrants further investigation.

The authors further show that MNGCs are distinct from macrophages by identifying unique immune cell types that intercalate specifically within MNGCs [8]. By analyzing protein-coding genes in MNGCs not shared with macrophages, Converse and colleagues identified that T-cells are closely associated with MNGCs. Specifically, CD3-positive T-cells cells negative for CD4 and CD8 (also referred to as double negative T-cells or dnTs) spatially integrate within MNGC networks. These dnTs, however, do not share cytoplasm with MNGCs, and are thus distinct cells within the broader MNGC network. While their physiological relevance in ovarian MNGCs remains unknown, their known roles in innate and adaptive immune capabilities continue to expand the functional breadth of MNGCs in aged ovaries.

In sum, results of this study contribute to our understanding of inflammaging processes within the ovary that appear conserved between aging mammals. The authors show that ovarian MNGCs are an important immune phenomenon distinct from other immune cell types and have functional relevance during reproductive aging. Further, the techniques and procedures utilized in these experiments to characterize ovarian MNGCs provide a detailed framework for investigating MNGCs in non-ovarian tissues. While questions remain as to the exact origin of MNGCs and the functional role of T-cells in MNGC networks, the study by Converse and colleagues aptly captures an exciting new area of ovarian aging research that is sure to influence how we view reproductive inflammaging and aging in general.

## Abbreviations

**:**
LCM: capture microdissection
MNGCs: multinucleated giant cells
NHP: nonhuman primate
